# Innovative approach of nomography application into an engineering educational context

**DOI:** 10.1371/journal.pone.0315426

**Published:** 2025-02-06

**Authors:** Trevor Blight, Pedro Martínez-Pagán, Leif Roschier, Daniel Boulet, Lorena Yepes-Bellver, Víctor Yepes

**Affiliations:** 1 Electrical Engineering, University of Adelaide, Adelaide, Australia; 2 Applied Near-Surface Geophysics Research Group, Departamento de Ingeniería Minera y Civil, Universidad Politécnica de Cartagena, Cartagena, Spain; 3 Helsinki University of Technology, Otaniemi, Finland; 4 Computer Technology Engineering, Red River College Polytechnic, Manitoba, Canada; 5 Mechanics of Continuous Media and Theory of Structures Department, Universitat Politècnica de València, València, Spain; 6 Institute of Concrete Science and Technology (ICITECH), Universitat Politècnica de València, València, Spain; Affiliated Hospital of Nanjing University of Chinese Medicine: Jiangsu Province Academy of Traditional Chinese Medicine, CHINA

## Abstract

Nomography is considered a branch of mathematics introduced by Maurice d’Ocagne in 1884 in France. The past century saw nomography grow as a graphical computing method used by scientists and engineers wishing to solve complex problems to a practical precision. Even though nomography has declined with the introduction of calculators and computers, it still offers potential in an educational setting. The recent development of open-source software is helping promote the use of nomograms among scholars in engineering courses who are aware of nomography’s capabilities. The main reason for this apparent and renewed interest in nomography is the capability of open-source software to generate customized and precise nomograms in seconds without the previously required mathematical background. In this work, we introduce Nomogen, a Python package able to build reliable and scalable 3-variable nomograms while avoiding past drawbacks such as manipulating determinants or manually drawing the scales. In this way, some nomograms generated by Nomogen have been tested on undergraduate and graduate students from different engineering backgrounds. Subsequently, a Likert scale survey was conducted, which showed that students had a great and renewed interest in nomography and found it helpful in the engineering learning process. Even though 78.4% of the respondent had never used nomograms, 86.5% believed that these analogical graphs allow a reasonable interpretation of the phenomenon when there are many variables, and, as a result, nomography with the assistance of open-source software, such as Nomogen or PyNomo, should be incorporated in the teaching process as part of their engineering education syllabus.

## Introduction

Nomography is a branch of mathematics in which projective transformations and different geometric methods are applied to create graphical representations of complex formulas and the inter-relationship among their variables [1]. The word nomography is the result of the combination of two Greek words: “*nomos*” (meaning “law”), and “*gramme*” (meaning “line”), which could be interpreted as the science of representing the law of mathematics by graphs or nomograms [2–4].

Nomography can be applied to many fields. They include: statistics [5], electronics [5], ballistics [6], heat transfer [5], radioactivity [5], medicine [7–9], mechanics [10, 11], forensics [12], energy [13, 14], food technology [5], engineering [15–19], physical and biological sciences [5], and business [5, 20]. In fact, during many decades throughout the twentieth century, there was a significant use of nomography, where many technical journals and books included nomograms that were time-saving in repetitive solutions of complex mathematical formulas [3, 21, 22]. Like slide rules, the appearance of more capable and powerful personal computers and handheld calculators toward the end of the twentieth century led to the disappearance of nomography instruction from most post-secondary institutions [2, 23]. Despite this, nomograms exist in published literature, such as design standards, handbooks, guides, manufacturers’ catalogs, etc. [24].

In nomography, the graphical representations are called nomograms or alignment charts [25]. These nomograms are graphical tools that consist of a system of scales that can be used to solve an equation by a geometrical construction, which can be performed by graphical elements such as a straightedge [1, 24]. Nomography still has important applications as a teaching tool, helping explain engineering topics through a clear visualization of complex formulas, which in turn deliver insights about how various values in the variables play out against each other. This is especially true when the correct values to choose for the calculations are uncertain, and the interplay between variables needs to be explored. Moreover, these graphical representations are reliable, intuitive tools capable of assisting users with limited mathematical background to solve engineering equations [23] accurately.

Open-source software can help construct nomograms with relative ease by eliminating the need to manually draw nomograms with precision and high detail [1, 24, 26]. Thus, using open-source software makes it possible to incorporate nomography into the learning syllabus of under-graduate engineering studies [27, 28]. In fact, as suggested by Kattan and Marasco [29], it is desirable to have a tool that could combine computerization with classical nomograms. In line with that statement, we present and examine the Python open source Nomogen package, which in conjunction with PyNomo package, can generate a neat PDF file containing the nomogram in high-quality vector graphics. PyNomo is a Python software package which handles the scaling and drawing of a nomogram [26, 30, 31].

This work introduces a 5-step approach to assess the student perceived significance of nomograms in engineering syllabus. This approach was conducted on a sample of 37 engineering students from two Spanish polytechnic universities, from under-graduate and graduate engineering courses. The main objectives of this tested approach were: (a) to assess the student opinion about the use of nomograms into the learning process; (b) to identify and classify those different opinions according to the type of engineering course and student age; (c) to evaluate the usefulness of nomograms into engineering teaching, their capabilities to help students in the understanding of multivariable equations and diminishing wrong results.

## A theoretical problem of nomograms

Any function of three variables, F(*u*,*v*,*w*), that can be expressed in the following form, known as the Massau’s determinant [3, 32, 33]:

$$|\begin{array}{ccc} g_{1}(u) & f_{1}(u) & 1\\ g_{2}(v) & f_{2}(v) & 1\\ g_{3}(w) & f_{3}(w) & 1 \end{array}|=0$$ (1)

Where *g*_1_(*u*), *f*_1_(*u*), etc., are any functions of the variables *u*, *v*, and *w*, then it is also possible to construct a nomogram consisting of three curves which are graduated respectively to represent the variables *u*, *v*, and *w*, and therefore computable by these means.

**Fig 1:**
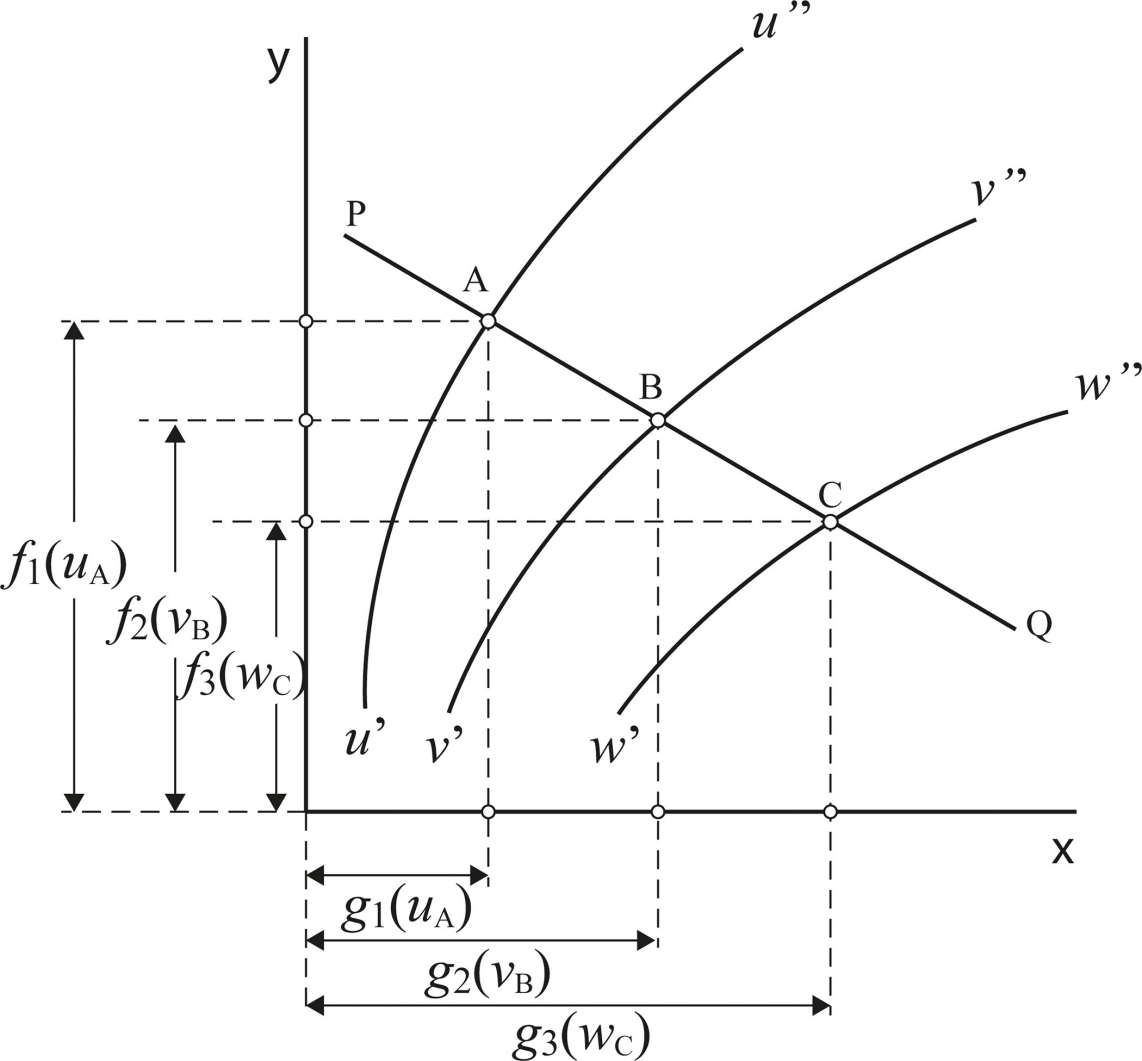
Nomogram for the variables *u*, *v*, and *w* that satisfies the Eq 1.

The way in which the above determinant forms the basis of such a nomogram is illustrated in Fig 1, from which it may be seen that the loci of *u*, *v*, and *w*, respectively, may be drawn by:

1. Plotting the positions of the points [*g*_1_(*u*) = x_1_, *f*_1_(*u*) = y_1_], [*g*_2_(*v*) = x_2_, *f*_2_(*v*) = y_2_], and [*g*_3_(*w*) = x_3_, *f*_3_(*w*) = y_3_], for various values of *u*, *v*, and *w*, between the limits for which the nomogram is to be constructed.
2. Marking each of the points obtained in this way with the corresponding values of *u*, *v*, or *w* and
3. Drawing lines through the various points representing u, v, and w respectively.

In Fig 1, the lines *u*’*u*”, *v*’*v*”, and *w*’*w*”, which result from this procedure are the required lines of the nomogram since it is obvious that any straight line PQ, drawn through two points A and B (corresponding to two known values, *u*_A_ and *v*_B_, of the variables *u* and *v*) will cut the line *w*’*w*” at a point C, corresponding to a value *w*_C_ of the variable *w* satisfying that the value of the determinant (Eq 1) is zero.

**Fig 2:**
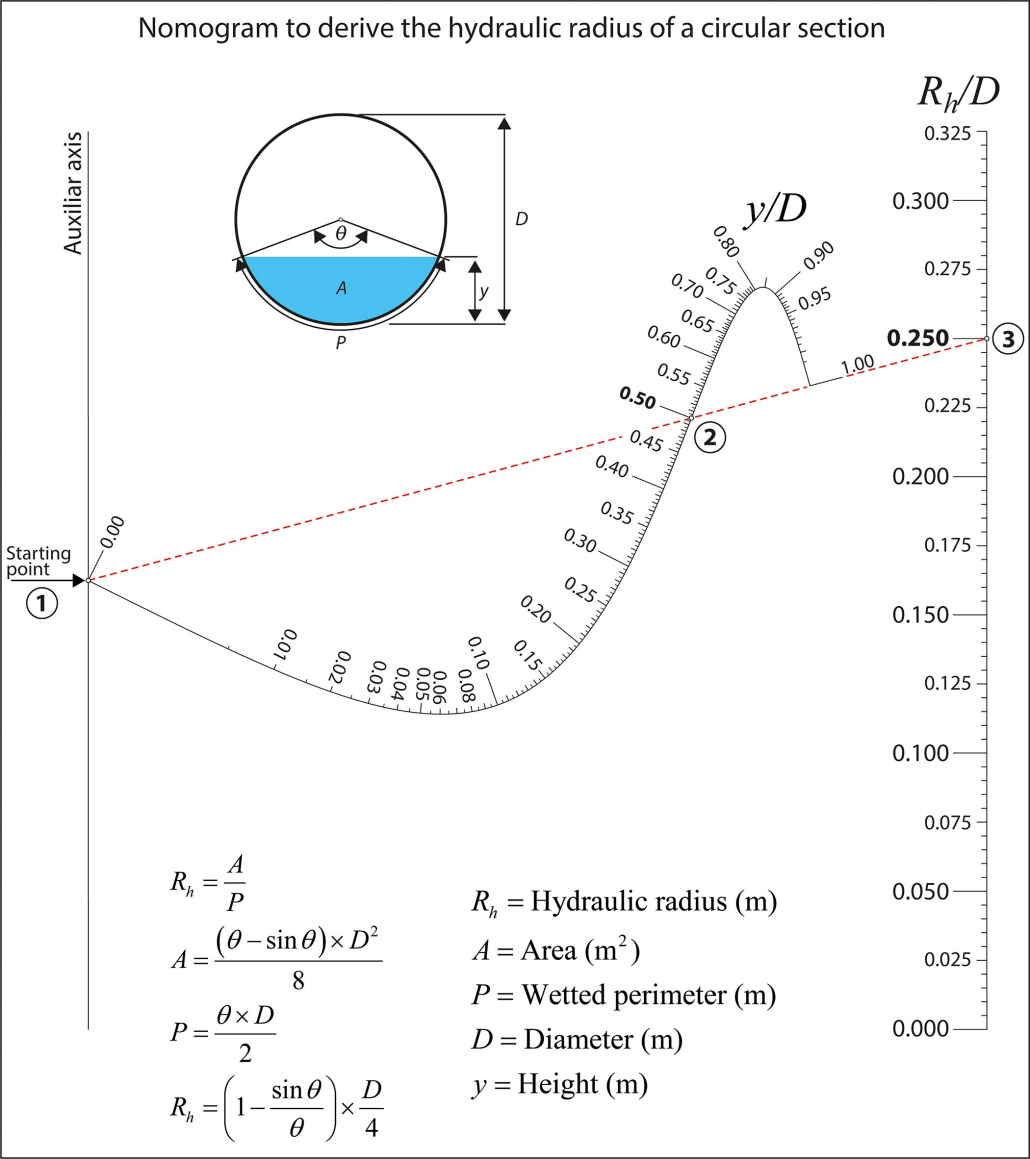
Example nomogram for deriving the hydraulic radius of a circular section.


Here below, we provide an expression for calculating the hydraulic radius of a circular section, where the nomogram associated (Fig 2) is a good example of how three-variable equations, even those with no analytical solution, can be modelled in a nomogram.

$$R_{h}=\frac{A}{P}$$ (2)

where *R*_*h*_ is the hydraulic radius, in m; *A* is the area of the circular section, in m^2^; and *P* is the wetted perimeter, in m.

However, it is not always obvious to see whether a given equation can be put under the Massau’s determinant form. Moreover, the manipulation of determinants requires certain skills and mathematic backgrounds, which not everyone possesses. Moreover, manually drawing reliable and precise scales for any nomogram is a very demanding task (Fig 2). The Nomogen package eliminates these drawbacks since it makes 3-variable nomograms fast and effortless to implement by auto-generating the scale lines from the associated function. More importantly, it does so without needing to know the associated determinant. These and other characteristics contribute to the introduction of nomograms in engineering-related undergraduate and postgraduate programs as complementary tools [27, 28].

In the next sections we introduce the Nomogen software pointing out its most relevant characteristics. We also provide some example nomograms generated with Nomogen to highlight its capabilities and potential contribution in an academic context as a teaching tool.

## Introducing the structure of Nomogen

Nomogen software, is a Python-based package designed to make a nomogram quick and easy by auto-generating the scale lines from a given function of three variables [34]. It is worth noting that a nomogram with M lines, N of them curves is known as Class M, Genus N [33]. Thus, Nomogen attempts to auto-generate Class 3, Genus 3 nomograms [33] relying on Python libraries to save written text files under suffix “.py” and to calculate the nomogram numerically using SciPy, NumPy, and PyNomo libraries [35]. In the process, the scale lines are represented by polynomials, after the polynomial degree has been established by Nomogen. At this point, it should be noted that smaller polynomial degree numbers make nomograms faster but less accurate. Also, the difference between PyNomo and Nomogen is that the former is a Python library for nomograms, which contains a module named Nomographer for drawing and scaling the curves of the nomogram’s scale lines, and Nomogen is another module that derives the equations for the curves automatically but needs the module Nomographer to draw the nomogram. Presently, Nomogen is not yet fully integrated into PyNomo.

**Fig 3:**
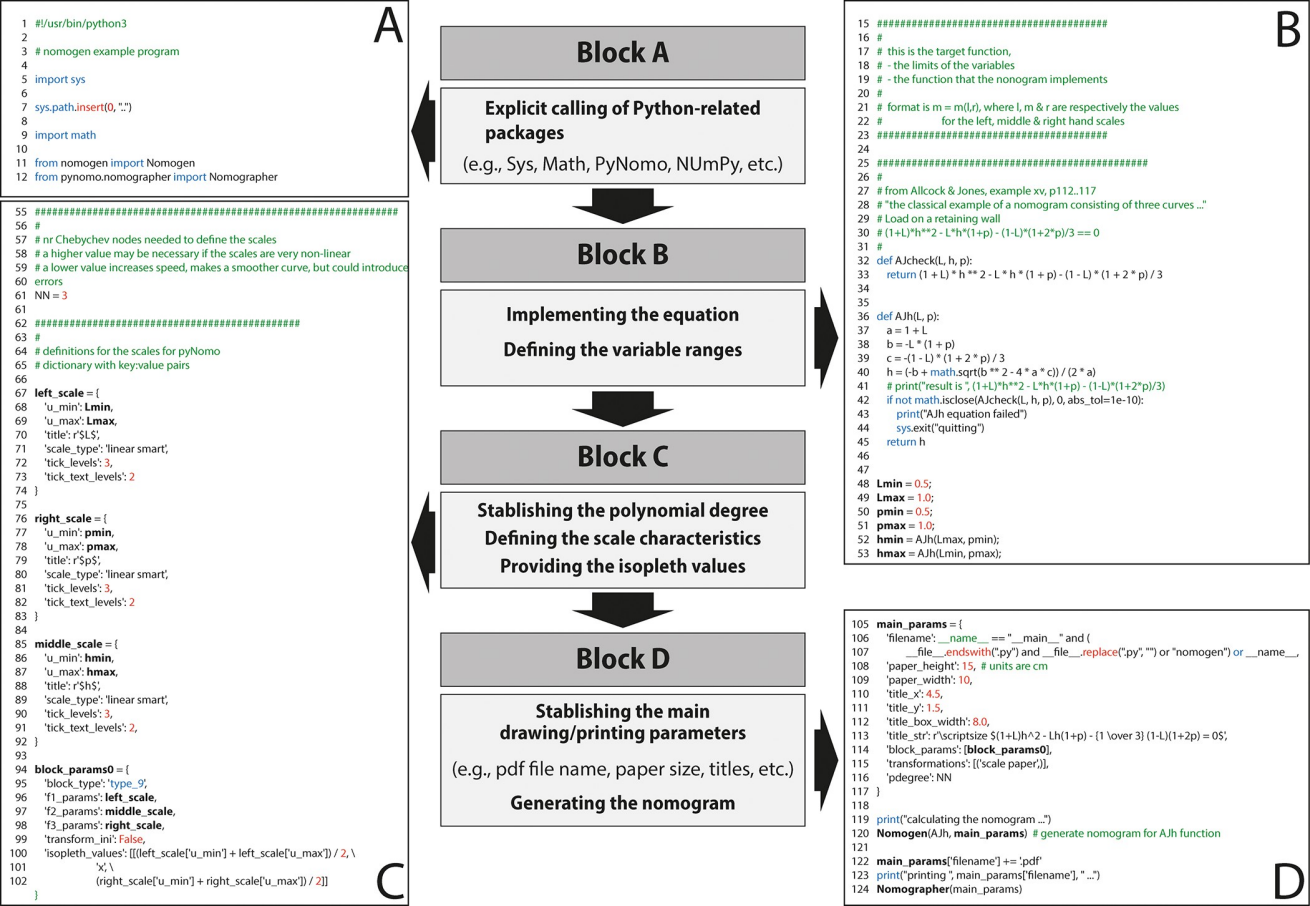
Screenshot showing the basic starting Nomogen’s code structure.

Let us examine a simple example step by step to demonstrate the basic Nomogen software structure and how the nomogram is generated. We will generate a nomogram that graphically solves the relative load on a retaining wall [33]. Consider Fig 3, which shows the common structure of Nomogen consisting in an initial code block “A” calling on Python-related packages such us Sys, Math, and PyNomo modules (Fig 3A).

Next, it follows the Block B which provides the equation defining the nomogram to be constructed (Fig 3B). Note that this example equation has the following format:

m=m(l,r) (3)

Where *l*, *m*, and *r* are, respectively, the values for the left-, middle-, and right-hand scales. In this example, we provide the equation relating the proportions of two retaining walls of the same height and designed to withstand the same pressure, one having vertical faces, and the other, one vertical and one sloping face. The formula is retrieved from Allcock and Jones [33], is as follows:

$$\left(1+L\right)\times h^{2}-L\times h\times \left(1+p\right)-\frac{1}{3}\times \left(1-L\right)\times \left(1+2\cdot p\right)=0$$ (4)

**Fig 4:**
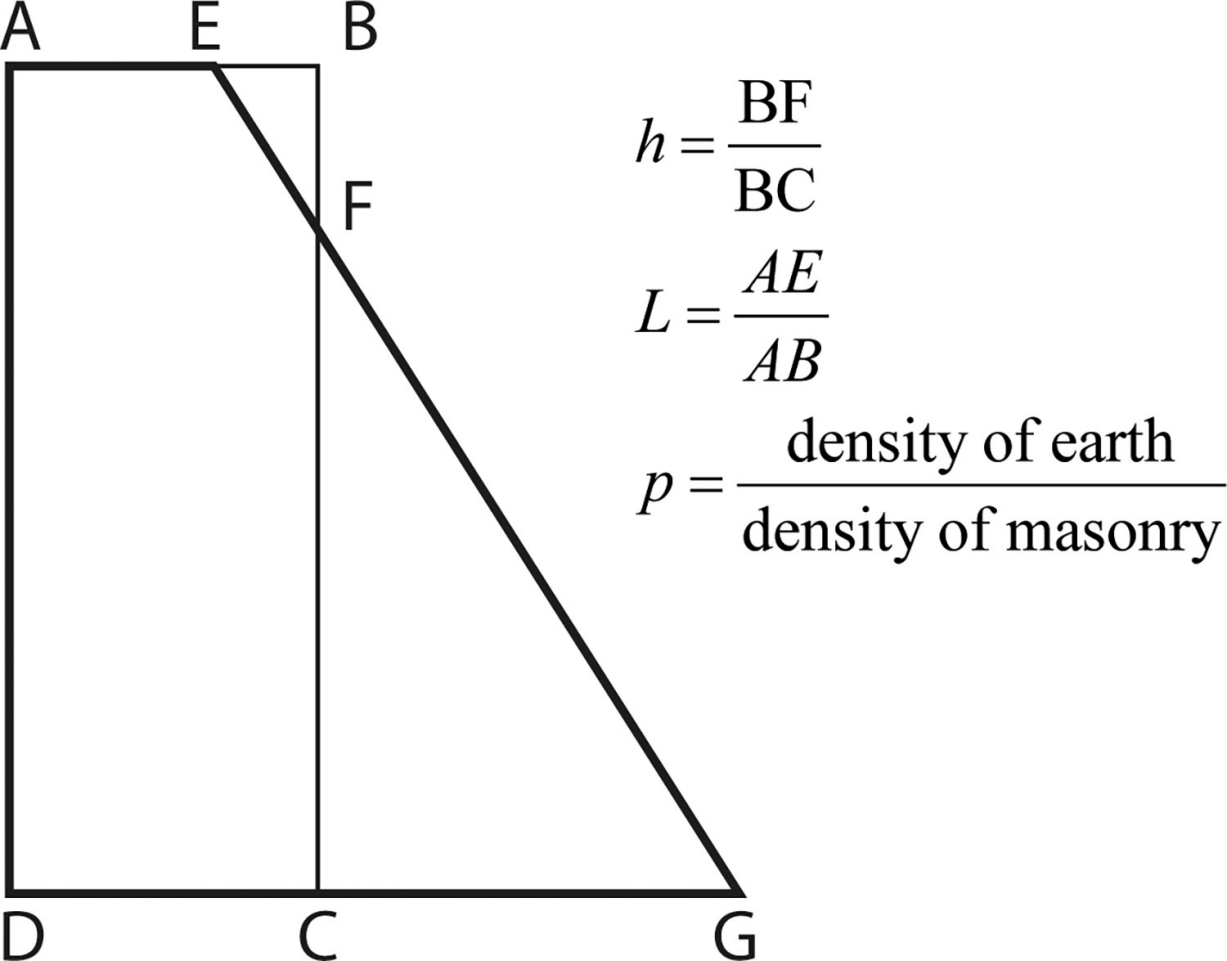
Variables in the Eq 4 for two retaining walls.

Consider Fig 4, which defines the variables employed in Eq 4, and where the two retaining walls are schematically represented.

Fig 3C presents the code employed for defining the parameters for each scale such as value range, title, scale type (log or linear), tick levels. These in turn define the marks or divisions on the scales, tick text levels and position the numbering. Moreover, the type of nomogram is specified in Block C. This will tell the PyNomo package what type of nomogram must be used. Since Nomogen solves equations of three variables expressed under their determinant form, these are specified as “type 9” nomograms. Block C also defines the two values that will be intersected by a straight line (the *isopleth*) enabling us to solve the value of the unknown third variable (middle scale). The isopleth drawing is optional since the nomogram may be constructed with or without it, however, the isopleth is useful for suggesting how the nomogram is used.

Lastly, in Block D, we specify the main nomogram parameters such as paper size, titles, type of transformations, number of points (“npoints”), etc. The PyNomo package will take in account the settings defined in this block generating the nomogram under pdf format (Fig 3D).

**Fig 5:**
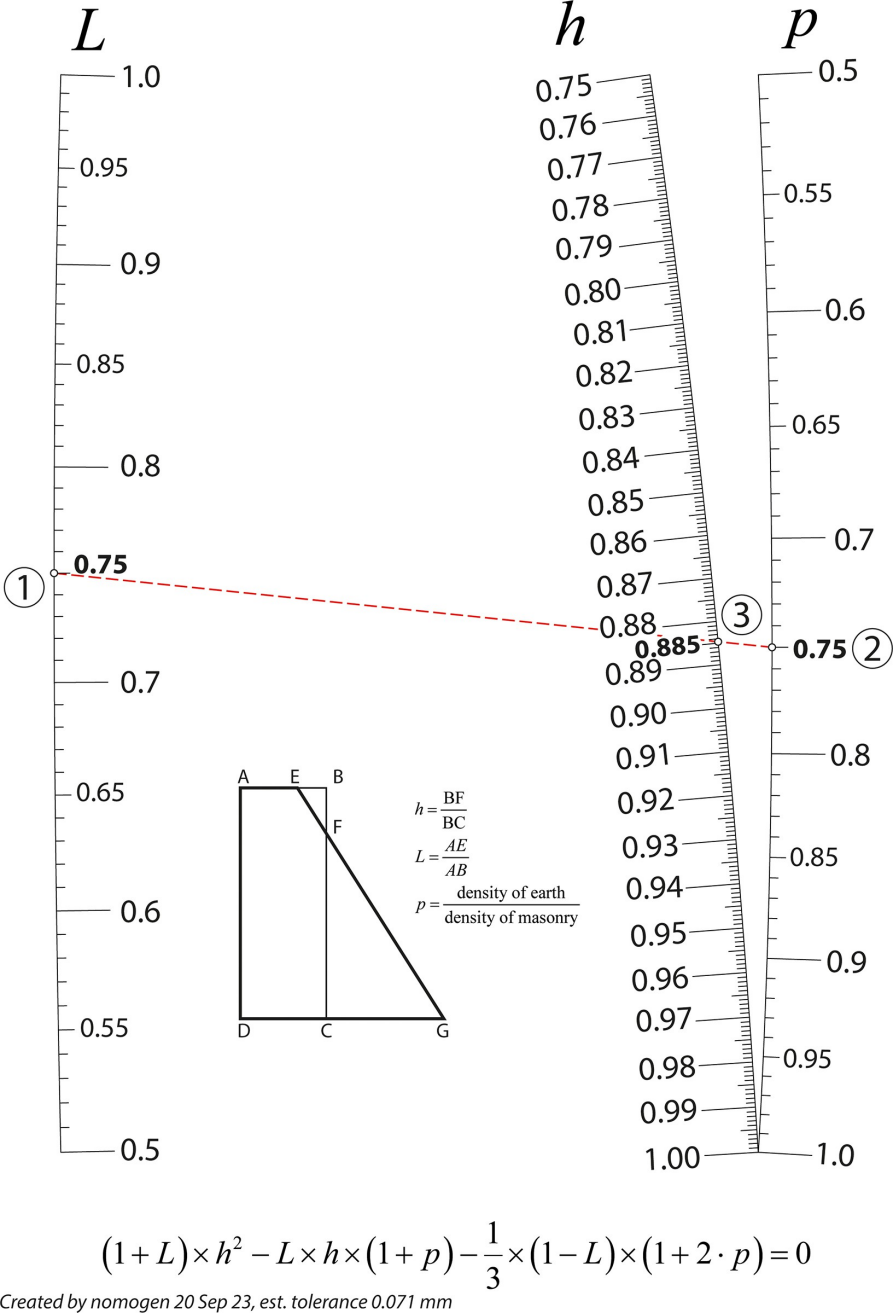
Nomogram obtained from the example.

Fig 5 depicts the nomogram finally generated by Nomogen with a tolerance or error of 0.071 mm. The tolerance value applies to the middle scale, and it indicates a sort of goodness of fit, or how closely does the nomogram implement the equation. If the error is unacceptable, the user can increase the polynomial degree to gain accuracy, or conversely if the accuracy is within acceptable limits, the polynomial degree can be decreased to gain speed of calculation. However, it is worth mentioning the tolerance is based on the specified size of the nomogram area–if the pdf is scaled up or down the value of the error becomes meaningless. Thus, Fig 5 provides a value of 0.885 for the variable *h*, assuming a value of 0.75 for the other variables *L* and *p*, by means of drawing the isopleth connecting those two values. It is worth noting that the nomogram created by Nomogen are in vector form so they can be edited for additional artwork in any graphic design software, which can support pdf and eps format, such as Adobe Illustrator, Microsoft Visio, LibreOffice Draw, or LaTeX. In fact, the nomogram in Fig 5 was edited using Adobe Illustrator for completing and adding additional details. At this point is worth adding that Nomogen calculates the curves by sampling the variables of scale lines of the nomogram at carefully chosen points. The number of points is chosen by the user–often 7 or 8 is a good choice. Then, these points are each assigned x and y coordinates by Nomogen. Moreover, the ends of the outer scale lines are tied to the corners of the nomogram’s defined area on the page to make the nomogram as large as possible. A least squares error is calculated for these points, and Python’s optimisation libraries are used to minimise this error and thus solve the nomogram. The curves are then interpolated from the final position of the points [34].

## Educational methodology

In this section we describe the educational methodology followed to assess the actual capabilities of nomography and its potential application as an educational tool for didactic purposes in an engineering learning environment.

This educational assessment was conducted on undergraduate and graduate engineering students at two Spanish polytechnic universities, enrolled on: (a) a 4-year mathematics and civil engineering degree (DMIC, according to its acronyms in Spanish), with a sample of 14 students at their second year courses; (b) a 2-year master on engineering of concrete (MIUH, according to its acronyms in Spanish), with a sample of 10 students at their first year courses; (c) a 4-year engineering in mineral resources and energy degree (GIRME, according to its acronyms in Spanish), with a sample of 6 students at their third year courses; (d) a 2-year master on civil engineering (MUICCP, according to its acronyms in Spanish), with a sample of 7 students at their second year courses. The choice of these groups was based on the variety of student profiles composing the samples, such as nationality, university, age, engineering background, etc. At this point, it should be noted that all the students involved in the implementation of this academic approach gave their consent orally, and all the information has been analysed anonymously.

In relation to that, these students could be grouped according to their age. Thus, the percentage of students younger than 22-year-old was 43.2%; the number of students, whose age ranged from 22-year-old to 24-year-old, corresponded to a percentage of 21.6%; the number of students, whose age ranged from 25-year-old to 30-year-old, corresponded to a percentage of 21.6%; and the percentage of students older than 30-year-old was 13.6%. Regarding their genre, a percentage of 62.2% were male and the remainder percentage of 37.8% were female, whereas in terms of nationality, a percentage of 73% corresponded to Spanish students, a 21.6% to Latin-American students, and the remaining percentage of 5.4% to other nationalities.

**Fig 6:**
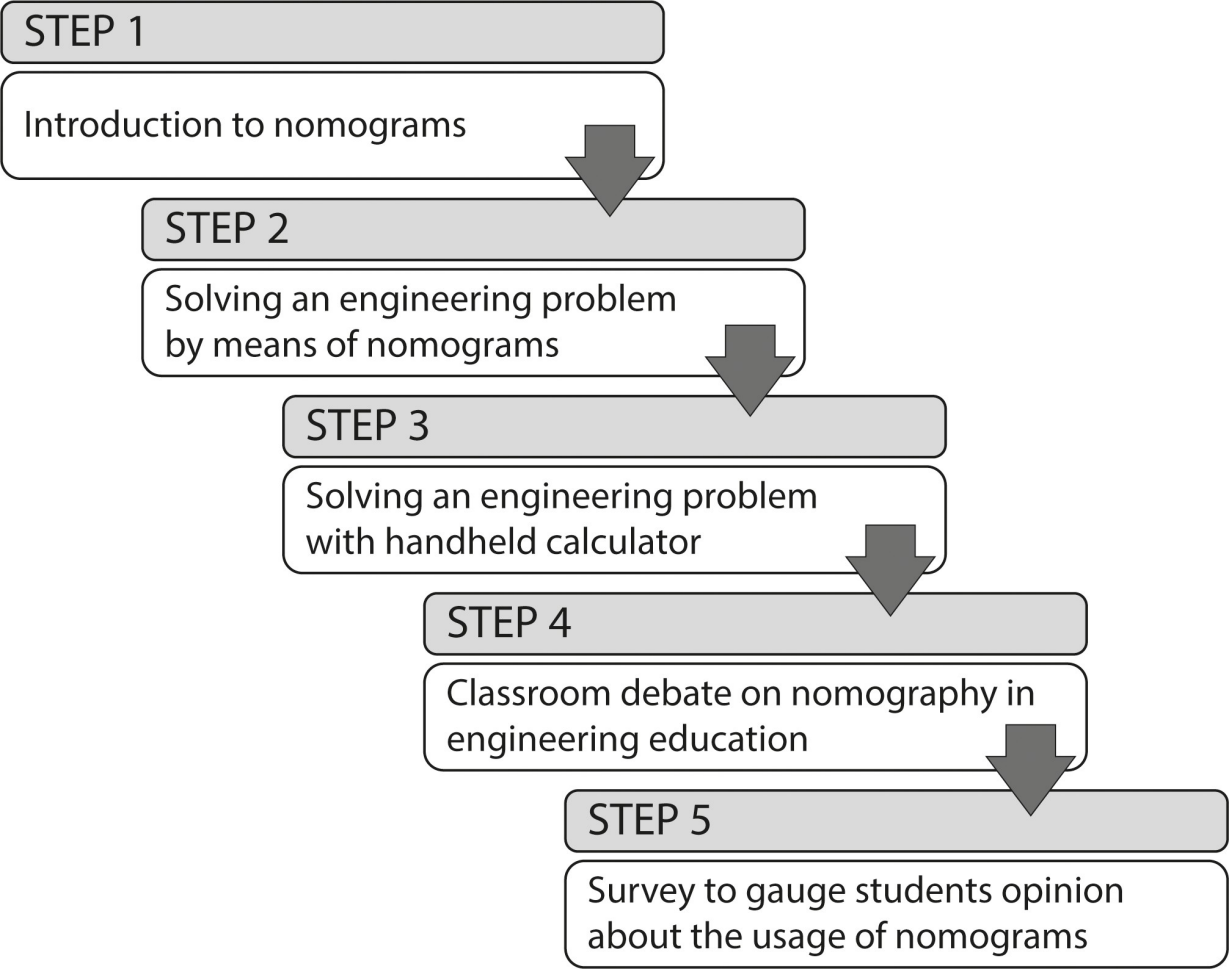
Diagram showing the 5-step approach to assess the student perceived importance of nomograms in academic context.

The applied educational methodology mainly consisted in five steps (Fig 6). In step 1, the teacher briefly introduced to the students the science of nomography, in which the main elements of the nomogram were described, some nomograms were examined, and students were trained to use them. In step 2, students had to solve an engineering problem using the nomograms associated with the equations without any additional assistance of handheld calculators. Then, in step 3, students had to solve the engineering problem of step 2, but this time using handheld calculators, and comparing the results with those obtained in step 2. In step 4, students were engaged in a debate on the advantages and disadvantages of nomograms, their degree of effectiveness, and the actual application in the academic context. Finally, in step 5, students took a survey, whose answers contributed to gauge the real impact of introducing the nomograms on learning programmes. Table 1 provides the questions used in the survey to evaluate the perceived advantages and disadvantages of nomograms among the students. The answer to each question followed the Likert scale: (a) strongly agree; (b) agree; (c) neutral; (d) disagree; and (e) strongly disagree.

**Table 1:** Questions used in the survey to evaluate the usefulness of nomograms.

No.	Question
P1	I have almost never used a nomogram in an academic or professional context
P2	I am not able to solve complex formulas without the assistance of a calculator
P3	If I do not have a close calculator, then a nomogram might be a useful tool
P4	A nomogram is useful for those people who lack a basic mathematical background
P5	A nomogram is useful for repetitive calculations
P6	Nowadays, a nomogram is useless because of the existence of more powerful handheld calculators and computers
P7	A nomogram allows a right interpretation of the phenomenon under study when too many variables are involved
P8	I think it is useful that teachers could use analogic tools, such as nomograms, in education context
P9	Nomograms are tools from the past and we should not spend any time with them

## Examples of nomograms for an engineering context

In this section, we provide some nomograms generated with Nomogen on topics frequently covered during courses taught in mining and civil engineering studies. These examples illustrate the actual capabilities of Nomogen for the relative effortless construction of alignment charts and their applications in under-graduate engineering studies. Thus, grasping the potentiality of this open-source tool, in conjunction with PyNomo software, for a high education academic environment. We select these examples from nomograms used in engineering studies to assist in solving engineering exercises. In these exercises students are asked to compare the results obtained with their handheld calculators and assess quickly how any subtle change in the value of the variables can impact on the final formula result as well as the validity of the formulas for the range considered.

### Nomogram for the determination of a hydrocyclone apex diameter

**Fig 7:**
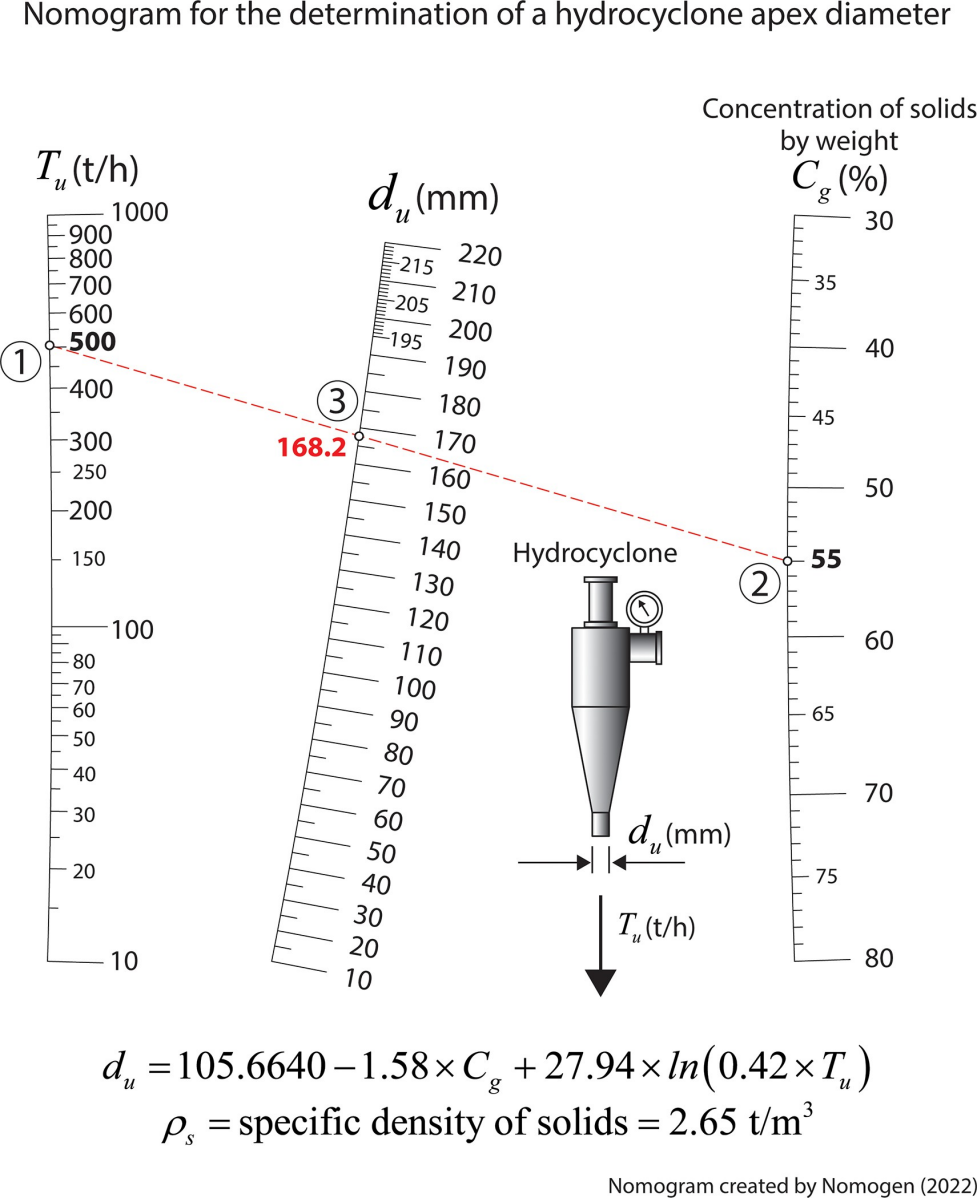
Nomogram for determining the hydrocyclone apex diameter in mining engineering studies.

Commonly, in mining engineering studies students are taught to design and size classifiers, which are installed in mineral processing operations. These include hydrocyclone classifiers which are conical devices for classification by centrifugal means of fine particles suspended in water. The coarser grains are collected at and discharged from the apex of the vessel while the finer particles are eliminated with the bulk of the water at the upper discharge orifice [36–38]. These units are essential in mining industry. Mining engineering students need to know how they operate and how to determine their operational variables. Specifically, students are asked to calculate the appropriate hydrocyclone apex diameter that will assure a proper unit operation. Thus, the expression that enables one to estimate the hydrocyclone apex diameter, assuming a specific density of solids of 2.65 t/m^3^, is given by [37, 38]:

$$d_{u}=105.6640-1.58\times C_{g}+27.94\times ln(0.42\times T_{u})$$ (5)

where *d*_*u*_ is the hydrocyclone apex diameter, in mm; *C*_*g*_ is the concentration of solids by weight, in percentage; *T*_*u*_ is the underflow rate, in t/h. The nomogram generated with Nomogen for educational purposes is shown in Fig 7. This nomogram allows obtaining the value of one unknown variable knowing the other two variables. In line with that, in Fig 7 depicts how the isopleth (dotted red line) connects two given values, 500 t/h and 55%, intersecting the middle *d*_*u*_ scale at the value of 168.2 mm, which is desired solution. This example demonstrates how Nomogen can easily generate curvilinear scales, something that would be very demanding if done manually as required in the past.

## Nomogram for determining the geometric burden in blast design

**Fig 8:**
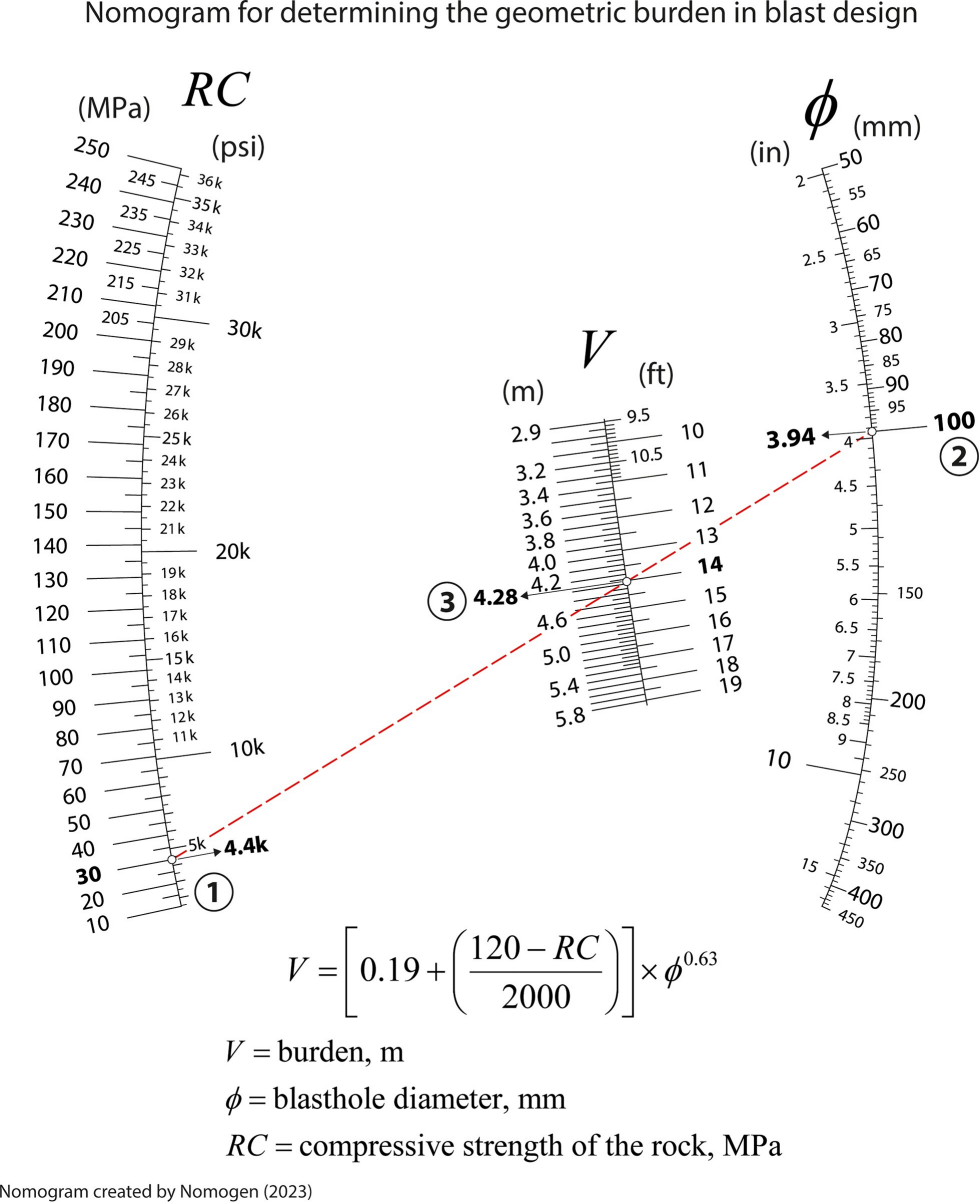
Nomogram for determining the geometric burden in blast design.

Blasting using explosives is a common operation in mining and civil works. However, efficient, and safe blasting techniques require a precise determination of all the parameters involved. These include the amount of explosive, the blasthole diameter, blastholes interspacing, burden, etc. [39]. Mining and civil engineering students are required to reliably determine these parameters. As part of their mining and civil and engineering studies, students are required to calculate the burden. The burden is a crucial parameter in blast design and is defined as the distance from the explosive charge located in a blasthole to the nearest free face of a mining exploitation or quarry [36, 39]. One of the expressions for estimating the required burden, commonly included in mining and civil engineering studies, is the following [40]:

$$V=[0.19+(\frac{120-RC}{2000})]\times \phi^{0.63}$$ (6)

where *V* is the burden, in m; *ϕ* is the blasthole diameter, in mm; and *RC* is the compressive strength of the rock, in MPa. The nomogram generated with Nomogen associated with Eq 6 can be seen in Fig 8. This nomogram proves its usefulness to quickly convert various engineering factors, constants, and symbols from the International System (SI) of units to other unit systems such as the Imperial System which remains in use among engineers and scientists in the USA among a few other countries. So, this could increase the educational value of nomograms in the classroom. Moreover, it is interesting to note that nomograms can be printed, etched or engraved on a metallic, plastic, or vinyl surface as required thus adding the capability to be employed in more difficult conditions as in work places.

### Nomogram for solving the Boltzmann expression

**Fig 9:**
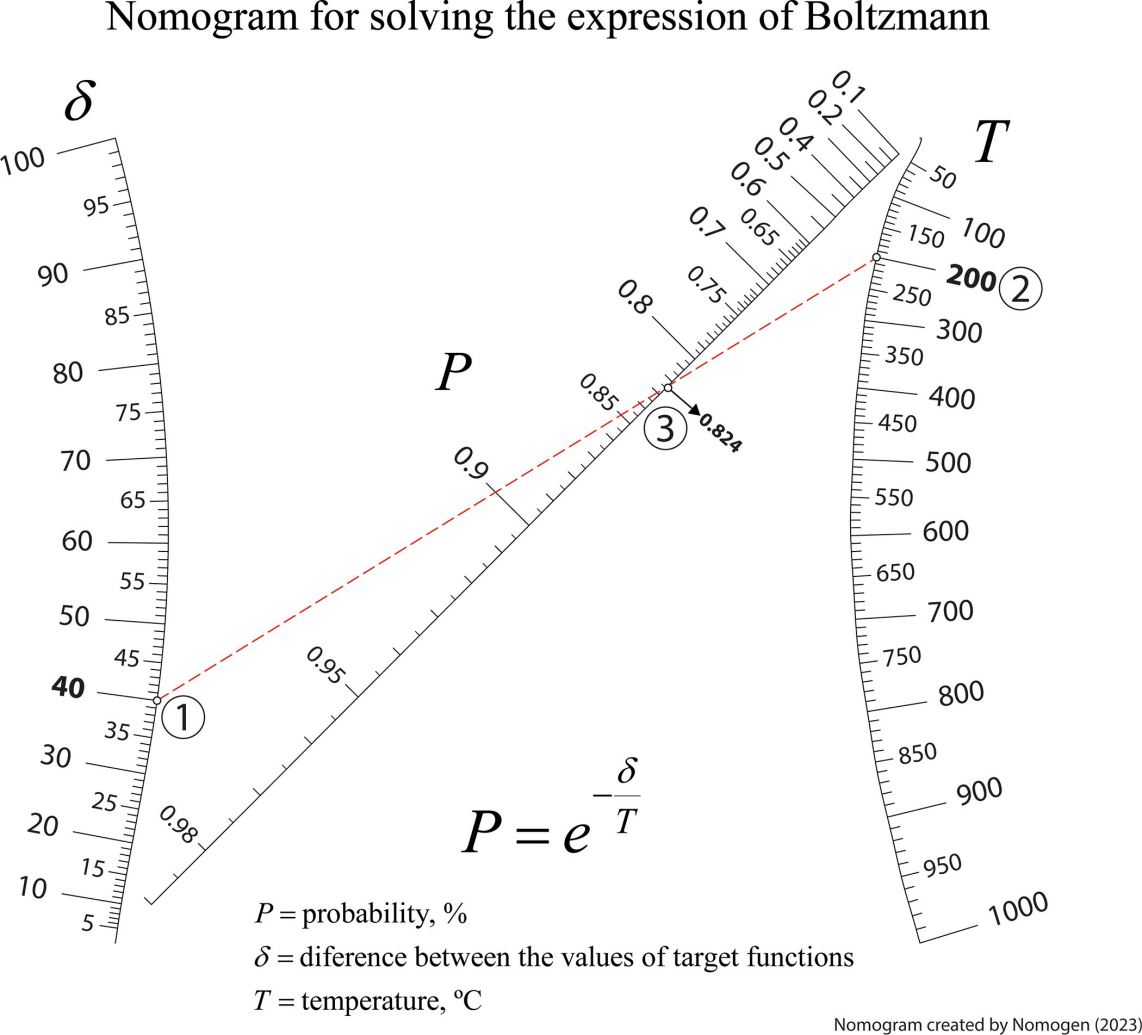
Nomogram for optimization analyses following the Boltzmann distribution.

In optimization studies, the physical concept of temperature is regarded as a parameter, denoted as *T*, that requires adjustment [41–43]. With regards to this, it is possible to establish parallels between processes that occur when molecules of a substance distribute themselves across different energy levels in pursuit of equilibrium at a specific temperature. Similarly, there are parallels between the minimization processes in local optimization (or maximization in its counterpart). In the optimization context, by setting the parameter *T*, a perturbation is introduced and directly accept the new solution if its cost decreases or with a probability proportional to the Boltzmann factor if it does not [41, 44]. So, in engineering studies there are many processes that follow the Boltzmann distribution, whose equation is:

$$P=e^{-\frac{\delta }{T}}$$ (7)

where *P* is the probability, in percentage; d is the difference between the values of target functions; and *T* is the temperature, in °C. Fig 9 shows the nomogram generated by Nomogen consisting in two curved scales and one straight scale in the middle. The isopleth in Fig 9 provides a value of 82.4% for the probability when the temperature is established in 200°C and assuming a difference between the values of target functions of 40.

### Nomogram for obtaining the average interest in machinery investment

**Fig 10:**
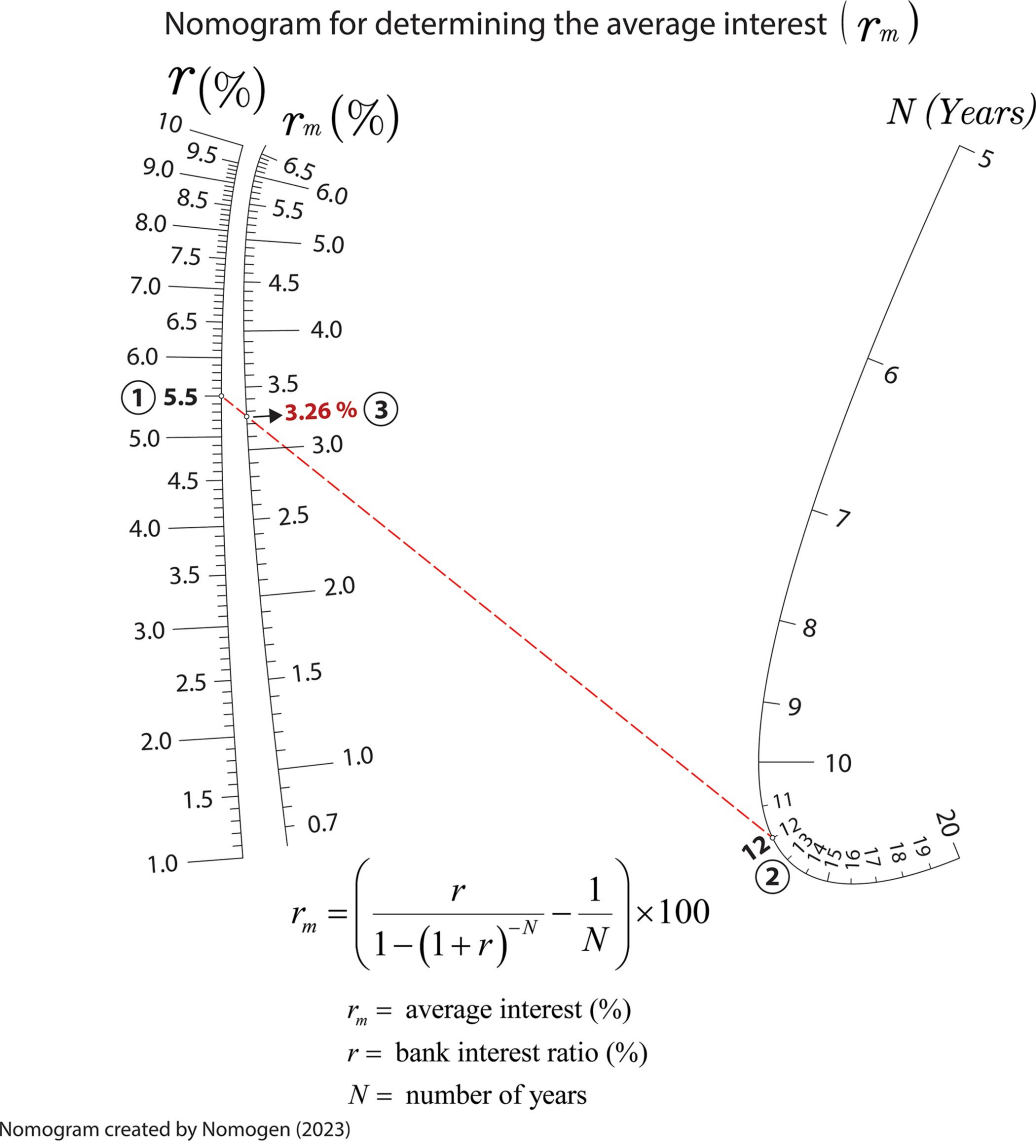
Nomogram for determining the average interest for a range of years going from 5 to 20.

**Fig 11:**
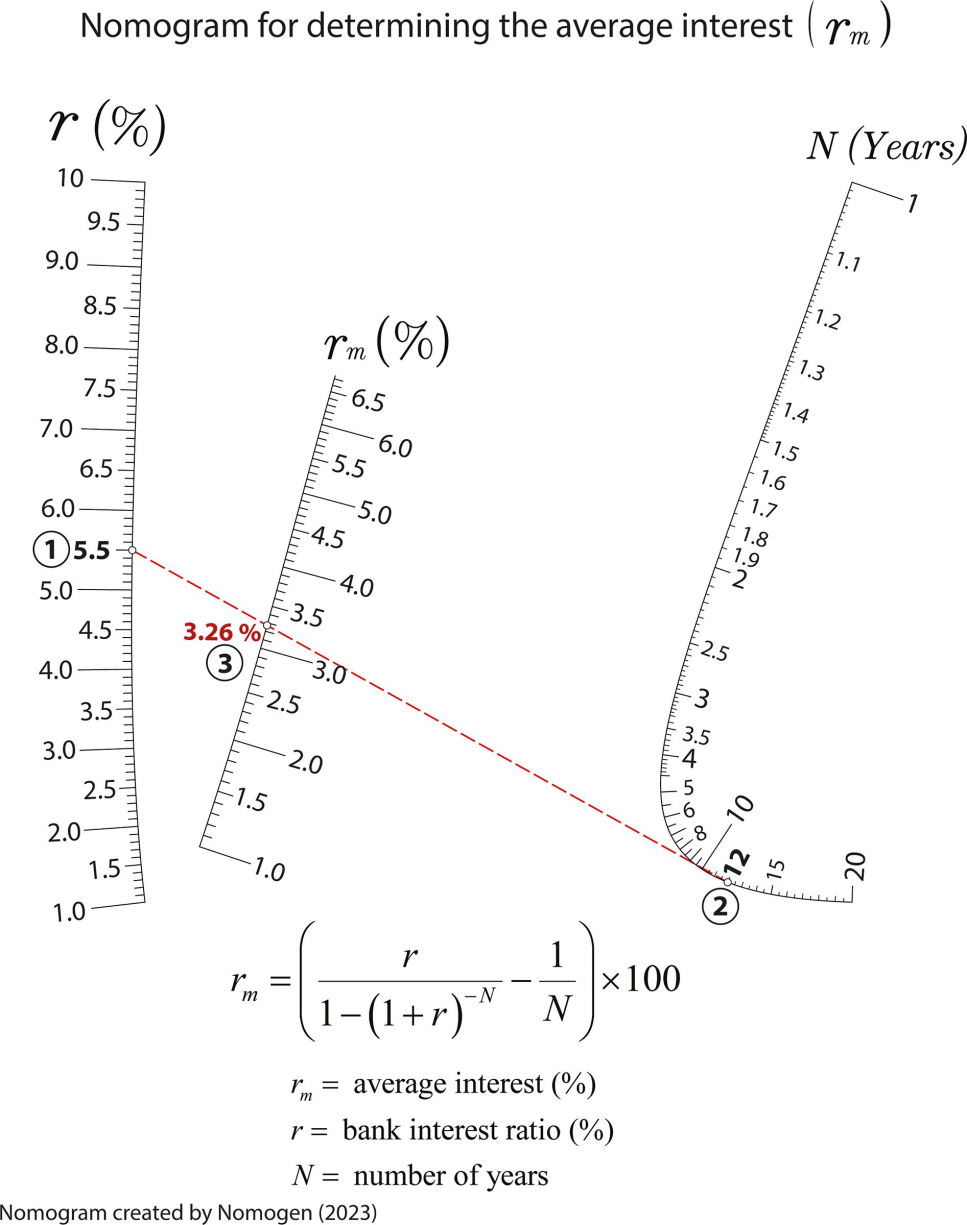
Nomogram for determining the average interest for a range of years going from 1 to 20.

Another common topic covered in engineering studies is the investment in machinery and its financing analysis. In this regard, students need to estimate the average interest associated with a machinery amortization period considering the amortization number of years, and the bank ratio. The equation to determine the average interest is:

$$r_{m}=(\frac{r}{1-{\left(1+r\right)}^{-N}}-\frac{1}{N})\times 100$$ (8)

where *r*_*m*_ is average interest rate, in percentage; *r* is the bank ratio; and *N* is the number of years for the machinery amortization. Figs 10 and 11 are the nomograms generated by Nomogen where the isopleth provides the value of the average interest assuming a bank interest ratio of 5.5%, and a period of amortization for the machinery of 12 years (*N*). These examples demonstrate how Nomogen can generate customized nomograms with fixed value ranges. This capability has educational interest as students can enlarge or zoom in on specific value ranges improving the accuracy in obtaining the variable values or define ranges appropriate to their own interest. In Figs 10 and 11, the range for the number of years has been chosen different affecting the drawing of the other scales and their readability. This shows how good nomograms are at seeing when something is wrong–sometimes it is unlikely to observe this from a spreadsheet or an equation.

## Results and discussion

Table 2 presents the mean and standard deviation values obtained for each question of the survey, from which it can be established that the estudents mostly agreed with the questions P3 and P8, on the contrary, they were in strong disagreement with the questions P6 and P9. On the other hand, regarding the questions P1 and P2, the estudents showed more dispersivity in their answers making them more difficult to group or to establish a representative result associated with their opinions. So, the survey provides evidence that the students are willing to use the nomograms in a educational context.

**Table 2:** Mean and standard deviation of the survey answers.

No.	Question	Mean	Std. Dev.
P3	If I do not have a close calculator, then a nomogram might be a useful tool	4.68	0.626
P8	I think it is useful that teachers could use analogic tools, such as nomograms, in education context	4.22	0.672
P1	I have almost never used a nomogram in an academic or professional context	4.16	1.302
P7	A nomogram allows a right interpretation of the phenomenon under study when too many variables are involved	4.14	0.787
P4	A nomogram is useful for those people who lack a basic mathematical background	4.11	0.906
P5	A nomogram is useful for repetitive calculations	3.86	1.159
P2	I am not able to solve complex formulas without the assistance of a calculator	3.51	1.239
P6	Nowadays, a nomogram is useless because of the existence of more powerful handheld calculators and computers	2.62	1.139
P9	Nomograms are tools from the past and we should not spend any time with them	1.97	0.799

Another interesting analyse conducted on the survey values was to statistically evaluate the established correlation among the answers. This analysis shows that, in general, there is not a strong correlation among the answers. The only remarkable correlation was obtained among the questions P8 and P9, for which the analysis provided a Pearson correlation coefficient value of -0.454 and a value of bilateral significance at level 0.01, meaning that the students agree with the idea that nomograms are not something from the past and, consequently, it is worth employing them in academia. In a similar way, the question P8 correlates quite well with the question P6, for which the analysis provided a Pearson correlation coefficient value of -0.398 and a value of bilateral significance at level 0.05, suggesting that, presently, nomograms are not useless when compared to handheld calculators and computers.

Moreover, a statistical Welch test was conducted, which demonstrates significant differences among the students opinions depending on what type of engineering course they are following to. This is especially true regarding the questions P1, with a Welch p-value of 0.004, P2, with a Welch p-value of 0.012, and P6, with a Welch p-value of 0.021, demostrating that students from MICCP courses are in more disagreement with the statement of question P1 “*I have almost never used a nomogram in an academic or professional context*” than the students from DMIC courses, whose opinion, in contrast, agrees with that statement. On the contrary, students from DMIC courses are in more disagreement with the statement of question P2 “*I am not able to solve complex formulas without the assistance of a calculator*” than the students from MICCP courses, whose opinion agrees with that statement. Finally, students from MICCP courses show less dissagreement with the statement of question P6 “*Nowadays*, *a nomogram is useless because of the existence of more powerful handheld calculators and computers*”. Also, the Welch test domonstrated the inexistence of differencies when the answers were grouped according to the student genre. On the contrary, significant differences were observed for the question P1 “*I have almost never used a nomogram in an academic or professional context*”, with a Welch p-value of 0.000, when the answers were grouped according to the students age, meaning that older students are more likely to have used nomograms.

## Conclusions

Nomography is a graphical computing method extensively used in the past by scientists and engineers. However, even today this computing method offers potential for educational purposes, especially in under-graduate and graduated studies. The main reason for this renewed interest in nomography in an academic context is the capability offered by Nomogen to quickly and easily generate customized and precise 3-scale nomograms without the need of the mathematical background normally required for this task.

In this paper, we briefly introduce Nomogen, a Python package that makes reliable and scalable 3-variable nomograms avoiding the drawbacks of the past such as the manipulation of determinants or manual drawing of the scales. In assisting this introduction, some nomograms are presented, which are associated with formulas selected from mining and civil engineering learning programs. These selected nomograms are part of learning activities in engineering programs led by the authors.

Also, this work presents an innovative 5-step approach for engineering learning contexts through the use of nomograms, generated by Nomogen software, to gauge the student’s opinion about the importance of using nomograms into engineering learning contexts. The degree of importance of nomograms in an academic context that is perceived by engineering students has been obtained through a survey conducted on undergraduate and graduate students at two different Spanish polytechnic universities. The students answers were statistically analyzed, whose results prove the usefulness and appropriateness of using nomograms as part of engineering syllabus. The surveyed students agree with the idea that nomograms are not something from the past and, consequently, it is worth employing them in academia. Also, the study has revealed that the opinion of engineering students can differ according to their engineering background or type of engineering course, thus, engineering students from final courses consider nomograms more useful than engineering students from initial courses.

This proposed methodology can be easily implemented into other educational contexts, not only in engineering but also in science studies. Lecturers can use this proposed approach basically consisting of an initial introduction to nomograms and, then, a practical activity with nomogram usage, allowing the students to visualize the relationships among their variables in conjunction with a better understanding of the formulas.

Finally, it can be stated that the use of nomograms in an academic context seems appropriate and they could become a valuable tool for accomplishing some learning autcomes in engineering universities.
